# An Inverse Relationship Between c-Kit/CD117 and mTOR Confers NK Cell Dysregulation Late After Severe Injury

**DOI:** 10.3389/fimmu.2020.01200

**Published:** 2020-06-25

**Authors:** Björn Bösken, Monika Hepner-Schefczyk, Sonja Vonderhagen, Marcel Dudda, Stefanie B. Flohé

**Affiliations:** Department of Trauma, Hand, and Reconstructive Surgery, University Hospital Essen, University Duisburg-Essen, Essen, Germany

**Keywords:** trauma, inflammation, IL-12 receptor, mTOR, functional reprogramming, natural killer cells, c-kit

## Abstract

Major trauma-induced tissue injury causes a dysregulation of the immune system. Severe systemic inflammation occurs early after the insult. Later on, an enhanced risk for life-threatening opportunistic infections develops that culminates at the end of the first week after trauma. CD56^bright^ Natural killer (NK) cells play a key role in the defense against infection due to their rapid release of Interferon (IFN) γ in response to Interleukin (IL) 12. NK cells are impaired in IFN-γ synthesis after severe injury due to a disturbed IL-12/IFN-γ axis. Thereby, a circulating factor mediates extrinsic suppression of NK cells. Yet unknown cell-intrinsic mechanisms manifest by day 8 after trauma and render NK cells unresponsive to stimulatory cytokines. In the present study, we investigated the origin of such late NK cell-intrinsic suppression after major trauma. Peripheral blood mononuclear cells (PBMC) were isolated from patients 8 day after severe injury and from healthy control subjects and were stimulated with inactivated *Staphylococcus aureus*. The expression of diverse cytokine receptors, intracellular signaling molecules, and the secretion of IFN-γ by CD56^bright^ NK cells were examined. After stimulation with *S. aureus*, NK cells from patients expressed enhanced levels of c-kit/CD117 that inversely correlated with IFN-γ synthesis and IL-12 receptor (IL-12R) β2 expression. Supplementation with IL-15 and inhibition of the transforming growth factor receptor (TGF-βR) I reduced CD117 expression and increased the level of IL-12Rβ2 and IFN-γ. NK cells from patients showed reduced phosphorylation of mammalian target of rapamycin (mTOR). Addition of IL-15 at least partly restored mTOR phosphorylation and increased IL-12Rβ2 expression. The reduced mTOR phosphorylation after severe injury was cell-intrinsic as it was not induced by serum factors. Inhibition of mTOR in purified NK cells from healthy donors by rapamycin decreased the synthesis of IFN-γ. Thus, impaired mTOR phosphorylation in response to a microbial challenge contributes to the cell-intrinsic mechanisms that underlie NK cell dysregulation after trauma. Restoration of the mTOR phosphorylation capacity along with inhibition of the TGF-βRI signaling in NK cells after severe injury might improve the immune defense against opportunistic infections.

## Introduction

Severe traumatic injury induces systemic inflammation that may cause early multi-organ damage. In parallel, an enhanced susceptibility to opportunistic infections develops that culminates at the end of the first week after injury and may persist even after discharge (1). The origin of the long-lasting suppression of the immune defense mechanisms after major trauma is only poorly understood (2, 3). Accordingly, effective therapeutic strategies that aim to restore immune homeostasis are lacking. Appropriate therapy of the immune dysregulation of injured patients is further complicated as the unbalance between inflammation and immunosuppression may shift to either side and at its best requires a personalized treatment (4).

Natural killer (NK) cells are cells of the innate immune system and play a central role in the defense against diverse infectious diseases and cancer (5). In human blood, two main populations of NK cells are distinguished: CD56^dim^ NK cells are highly cytotoxic and may kill cells infected with viruses or tumor cells. CD56^bright^ NK cells are potent in the secretion of cytokines such as Interferon (IFN) γ that is required for the activation of macrophages and dendritic cells (DCs) during the elimination of bacterial infection (6, 7). Interleukin (IL) 12 is released by monocytes/macrophages and DCs upon contact with microbial components and stimulates NK cells for IFN-γ synthesis (8, 9). The IL-12 receptor (IL12R) consists of a constitutively expressed β1 and an induced β2 chain. Binding of IL-12 to its receptor induces the phosphorylation of Signal Transducer and Activator of Transcription (STAT) 4 that translocates into the nucleus where it enables the transcription of the *IFNG* gene (10, 11). The T-box transcription factor T-bet cooperates with STAT4 in *IFNG* gene transcription and additionally promotes *IL12RB2* gene transcription (12). The cytokines IL-2 and IL-15 increase the IL-12-induced IFN-γ synthesis by NK cells in a synergistic manner (13, 14).

NK cells express both T-box transcription factors T-bet and Eomesodermin (EOMES) and thereby may be distinguished from innate lymphoid cells (15). A part of circulating CD56^bright^ NK cells expresses the tyrosine kinase CD117 (also known as c-kit) that was originally associated with the phenotype of NK cell progenitors (16, 17).

Considering the relevance of NK cells in immune defense it is apparent that NK cells might be involved in the immune dysregulation after major injury. A recent study followed total NK cells for 5 d after trauma and observed a transient decrease in the expression of T-bet and IFN-γ (18). We have previously shown that CD56^bright^ NK cells are rapidly and long-lasting suppressed after major trauma in terms of IFN-γ synthesis in response to *Staphylococcus aureus*, a frequent cause of opportunistic infections after injury (19). We identified an impaired IL-12Rβ2 expression that was associated with decreased STAT4 activation and IFN-γ synthesis. Although NK cells were similarly suppressed in IFN-γ synthesis from 24 h to at least 4 weeks after injury there were qualitative differences in the underlying mechanisms: extrinsic suppression of NK cells occurs early after injury and is mediated by a soluble factor that signals through the transforming growth factor (TGF) β receptor (TGF-βR) I. In addition, so far unknown endogenous changes establish in NK cells between 6 and 8 day after trauma that impair the IL-12/IFN-γ axis independent of the suppressive factor in the serum (19). Thus, the endogenous changes in NK cells overlap with the reported time window of cumulating infectious complications after trauma. In the present study, we aimed to shed light on the endogenous mechanisms in NK cells that arise late after traumatic injury and contribute to the impaired IFN-γ synthesis in the context of *S. aureus* infection.

## Materials and Methods

### Study Design and Patients

Severely injured patients (Injury Severity Score ≥16; age ≥18 years) who were admitted to the emergency room of the Department of Trauma, Hand and Reconstructive Surgery of the University Hospital Essen between August 2017 and September 2018 were included after approval by an independent physician. Exclusion criteria were isolated head injury, immunosuppressive therapies, cancer, and autoimmune diseases. Serum and heparinized blood samples were obtained from *n* = 14 patients 8 day after trauma. Blood from sex and age matched healthy donors was drawn as controls. The patient characteristics are shown in Supplementary Table 1.

The study was approved by the local ethic committee of the University Hospital Essen and written informed consent was obtained from patients or their legal representatives and from healthy donors. The study was conducted according to the Declaration of Helsinki.

### Isolation of Mononuclear Cells and Preparation of Serum

Peripheral blood mononuclear cells (PBMC) were isolated from heparinized blood by Ficoll density gradient centrifugation and subsequent red blood cell lysis (Sigma-Aldrich, Taufkirchen, Germany). PBMCs were used for cell culture or immediately stained for FACS analysis. Serum was obtained from clotted whole blood after centrifugation at 2,000 g for 10 min and immediately used or stored at −20°C for further analysis.

### Cell Culture

PBMC were cultured in VLE RPMI 1640 Medium (containing stable glutamine; Biochrom, Berlin, Germany) supplemented with 100 U/ml Penicillin and 100 μg/ml Streptomycin (Sigma-Aldrich Chemie, Taufkirchen, Germany) and 10% autologous serum.

4 × 10^5^ cells/well were cultured in 96-well flat bottom plates (BD Biosciences, Heidelberg, Germany) in a total volume of 200 μl/well and incubated at 37 °C and 5% CO_2_ in a humidified atmosphere.

After 1 h rest, PBMC were stimulated with heat-killed *S. aureus* (10^6^ bacteria /ml; Invivogen, San Diego, CA). Eighteen hour later, the cells were harvested for FACS analysis. Where indicated, 4 μM SB431542 (inhibitor of ALK4, ALK5, and ALK7; Tocris Bioscience, Bristol, UK), 5 ng/ml recombinant human IL-15 (PeproTech, Hamburg, Germany), or a combination of both was added to the cells before stimulation with the bacteria.

For the preparation of “conditioned medium,” PBMC were cultured in 2% FCS and stimulated with heat-killed *S. aureus* (0.5 × 10^6^ bacteria /ml). Supernatants were harvested after 18 h.

### NK Cell Assay

NK cells were isolated from PBMC of healthy donors using the “Human NK cell isolation kit” (Miltenyi Biotec, Bergisch Gladbach, Germany) according to the manufacturer's protocol. NK cells were seeded in 96-well plates (2 × 10^4^/well) in medium supplemented with 5% serum from healthy donors. Conditioned medium from PBMC was added at 25% v/v. The mTOR inhibitor rapamycin (2 nM; PeproTech, Hamburg, Germany) or its solvent (DMSO) was added. Eighteen hour later, the cells were harvested for FACS analyses.

### Flow Cytometry

Three color staining of cell surface molecules was performed as described previously (19) using antibodies against CD3 (clone MEM-57, FITC-labeled, ImmunoTools, Friesoythe, Germany) and CD56 (clone CMSSB, APC-labeled, Thermo Fisher Scientific, Waltham, MA) in combination with one of the following PE-labeled antibodies: anti-IL-12Rβ2 (clone REA333, Miltenyi Biotec), anti-CD94 (clone DX22, BioLegend, San Diego, CA), anti-CD122 (clone TU27, BioLegend), anti-CD132 (clone TUGh4, BioLegend). Where indicated PE-Cy7-labeled antibodies against CD117 (clone 104D2, BioLegend) was used as a fourth color.

Intracellular staining of IFN-γ was performed as described previously (19) using antibodies against IFN-γ (clone 4S.B3, PE-labeled, BioLegend) in combination with anti-CD3 and anti-CD56. Intracellular staining of mTOR, EOMES, and T-bet was performed using the “FoxP3/Transcription Factor Staining Buffer Set” (Thermo Fisher Scientific) according to the manufacturer's instructions. After surface staining with anti-CD3 and anti-CD56 as described above, the cells were fixed and permeabilized before staining with PE-labeled antibodies (all from eBioscience, Thermo Fisher Scientific) against T-bet (clone 4B10), EOMES (clone WD1928), or mTOR (clone MRRBY). For all stainings appropriate isotype control antibodies were used to determine the threshold of positive staining.

Data were acquired using a FACSCalibur (BD Biosciences; Franklin Lakes, NJ) and analyzed using NovoExpress software (ACEA Biosciences, San Diego, CA). The expression of respective molecules was determined on gated CD3^−^CD56^bright^ NK cells.

Due to technical failure or an insufficient number of PBMC after isolation from whole blood it was unfeasible to generate all data from all patients.

### Statistical Analyses

Statistical analysis and graphical presentation were performed using GraphPad Prism Version 5 software (GraphPad Software, La Jolla, CA). The non-parametric Mann-Whitney *U*-test and Wilcoxon signed rank test were used for statistical analysis as depicted in the figure legends. Spearman *r* analysis was used to test the correlation between two parameters.

## Results

### CD3^−^CD56^bright^ Cells Express Characteristic Markers of Differentiated NK Cells Late After Trauma

We included *n* = 14 severely injured patients and *n* = 14 age- and sex-matched healthy controls in our study (patient characteristics are listed in Supplementary Table 1). On day 8 after trauma, the patients displayed elevated levels of C-reactive protein but normal levels of procalcitonin. Twenty-one percent of the patients developed sepsis that was diagnosed beyond day 8.

**Figure 1:**
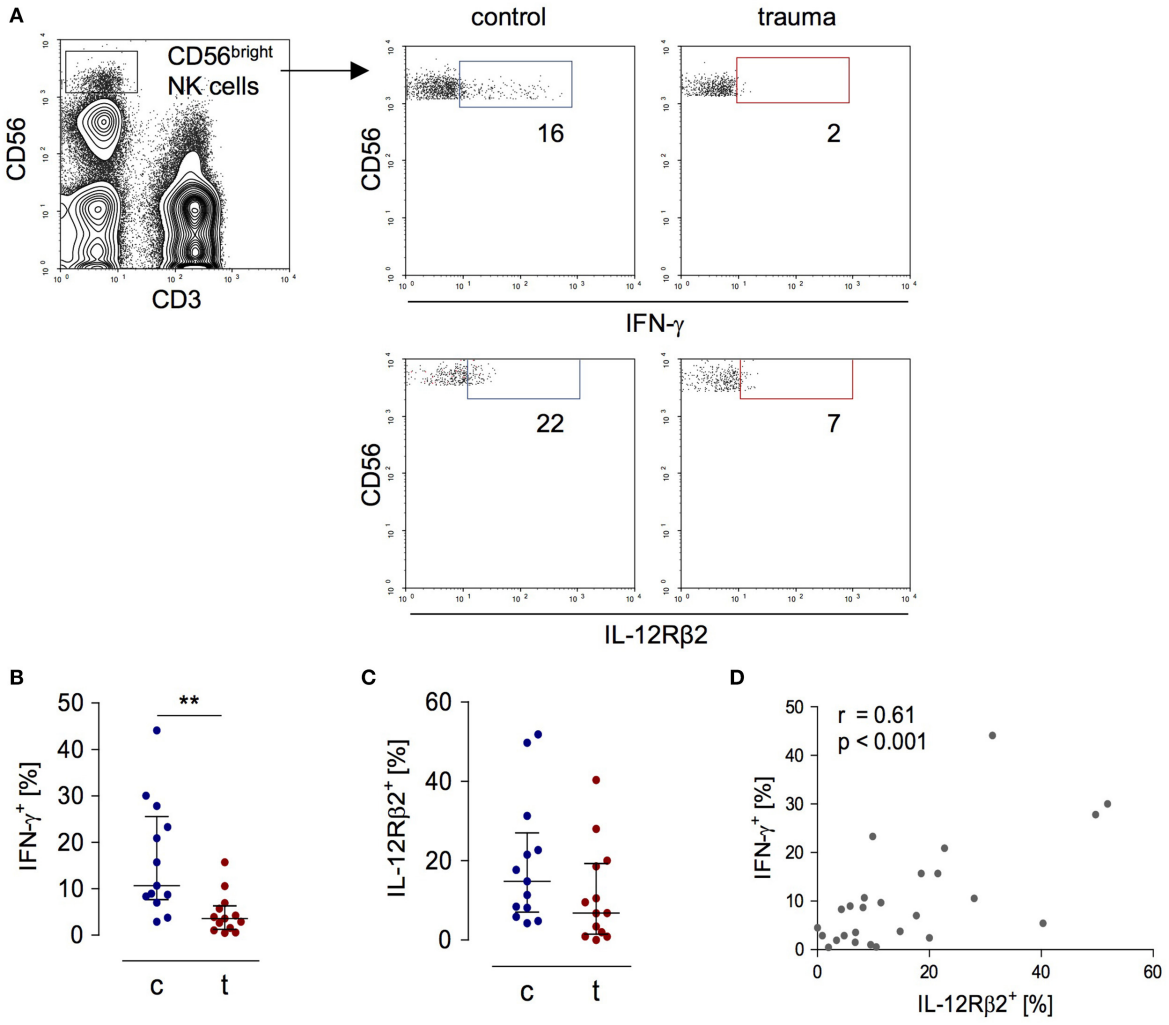
Impaired IFN-γ synthesis of CD56^bright^ NK cells after severe trauma correlates with reduced expression of the IL-12Rβ2 chain. PBMCs were isolated from healthy donors (c) and trauma patients (t) 8 day after trauma. Cells were exposed to heat-killed *S. aureus*. After 18 h, the IFN-γ synthesis and IL-12Rβ2 expression were determined by flow cytometry. **(A)** Gating strategy of CD3^−^CD56^bright^ NK cells. Representative dot plots are shown. Numbers indicate the percentage of positive cells. **(B,C)** Cumulative results of the percentage of IFN-γ^+^ cells (**B**; *n* = 13) and IL-12Rβ2^+^ cells (**C**; *n* = 13) among CD56^bright^ NK cells. Horizontal lines indicate the median/interquartile range. Statistical differences between control and trauma were tested using the Mann-Whitney *U*-test. **(D)** Spearman correlation between the percentage of IL-12Rβ2^+^ and IFN-γ^+^ CD56^bright^ NK cells. **p < 0.01.

In order to evaluate the activity of CD56^bright^ NK cells, PBMC obtained from patients 8 day after trauma and from healthy control subjects were stimulated with inactivated *S. aureus* bacteria and the expression of IFN-γ and of the IL-12Rβ2 chain by CD3^−^CD56^bright^ NK cells was determined by flow cytometry (for gating see Figure 1A). For our study, we used flow cytometry because it allows the analysis of surface and intracellular protein expression on and in selected cell subpopulations. As expected, NK cells from trauma patients displayed diminished levels of IFN-γ and IL-12Rβ2 (Figures 1B,C). The expression of IFN-γ correlated with the expression of the IL-12Rβ2 chain (Figure 1D).

**Figure 2:**
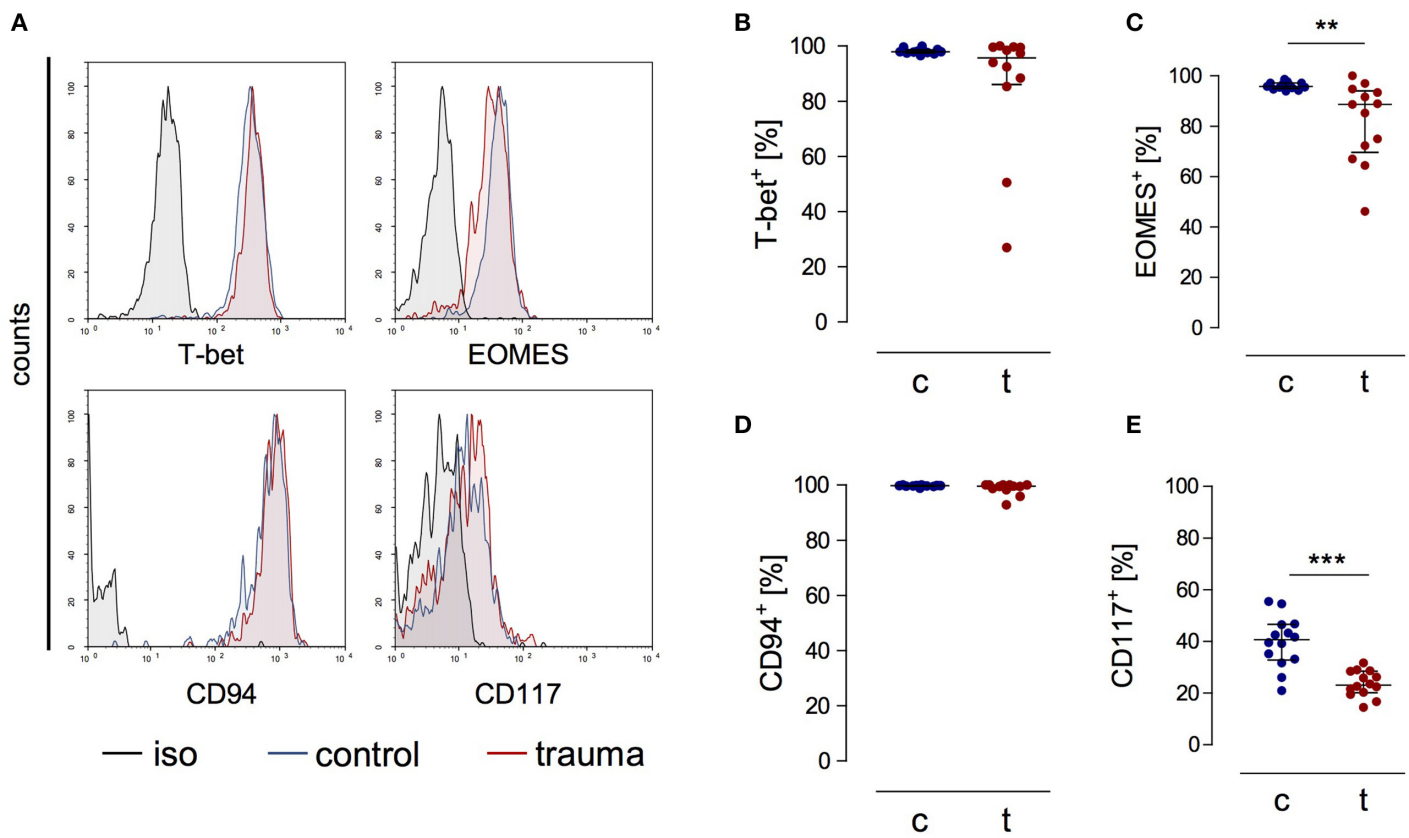
CD56^bright^ NK cells from trauma patients express characteristic markers of mature NK cells. PBMCs from control subjects (c) and trauma patients (t) were stained *ex vivo* for the expression of T-bet, EOMES, CD94, and CD117. CD3^−^CD56^bright^ NK cells were gated. **(A)** Representative histograms. **(B–E)** Cumulative data on the expression of T-bet (**B**; *n* = 12), EOMES (**C**; *n* = 13), CD94 (**D**; *n* = 12), and CD117 (**E**; *n* = 14). Horizontal lines indicate the median/interquartile range. Statistical differences between control and trauma were tested using the Mann-Whitney *U*-test. **p < 0.01 and ***p < 0.001. iso, isotype control.

CD3^−^CD56^bright^ cells from patients expressed the transcription factors T-bet and EOMES as well as CD94 *ex vivo* that are all characteristic markers for NK cells [(20); Figures 2A–D]. CD56^bright^ NK cells moreover expressed the β and γ chains of the IL-15 receptor comparable to NK cells from controls (Supplementary Figure 1). The percentage of NK cells that expressed CD117, a marker that has been linked with NK cell progenitors, was decreased after trauma (Figure 2E). Thus, despite their reduced capacity to secrete IFN-γ late after trauma CD3^−^CD56^bright^ cells display a phenotype of mature NK cells similar to NK cells from controls.

### Expression of CD117 Is Linked With the Suppressed Cytokine Release of NK Cells After Trauma

**Figure 3:**
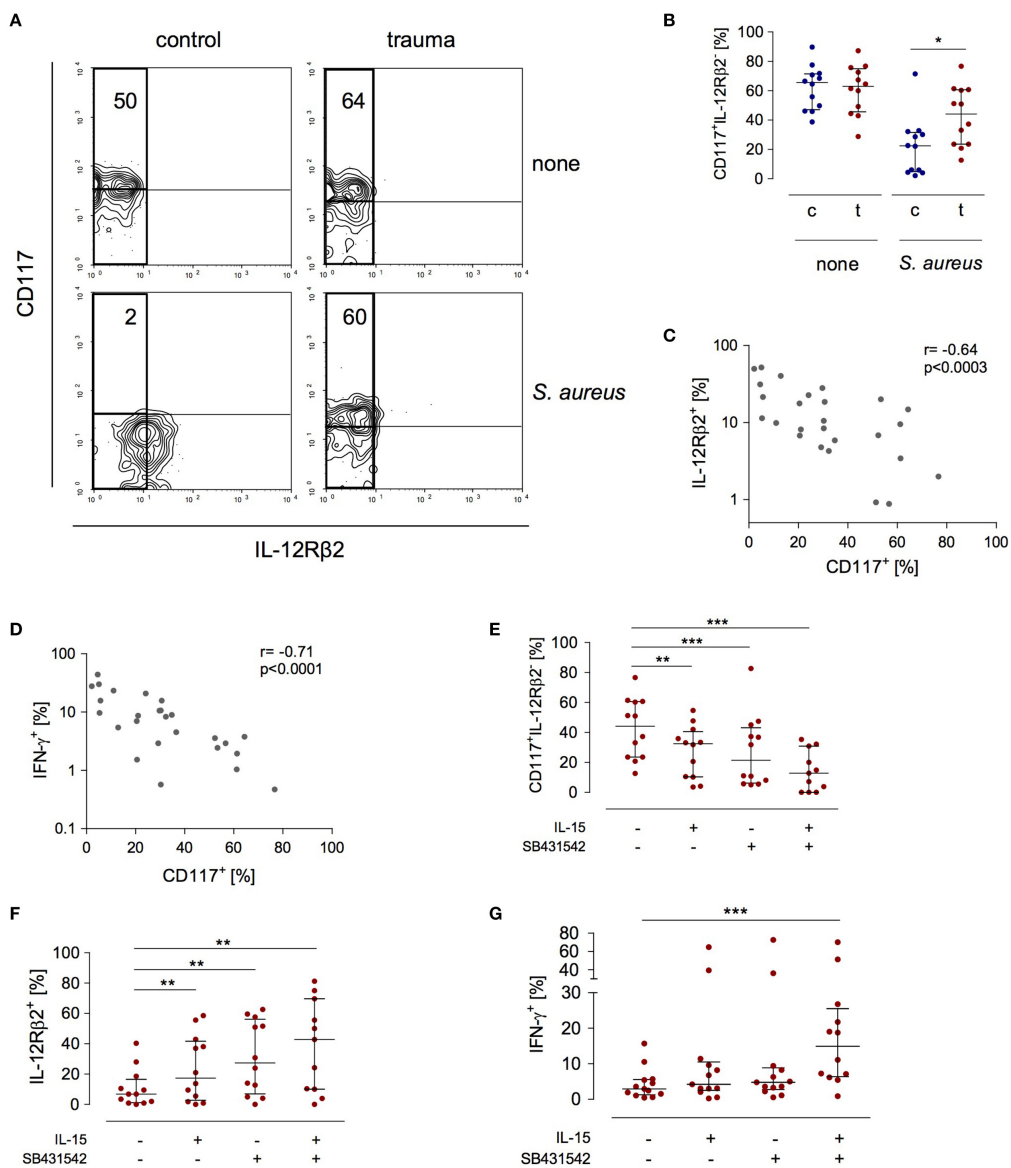
Stimulated CD56^bright^ NK cells express increased levels of CD117 after trauma that inversely correlates with IFN-γ synthesis and IL-12Rβ2 expression. PBMC from control subjects (c) or patients (t) were stimulated with *S. aureus* and the expression of CD117, IFN-γ, and the IL-12Rβ2 chain was examined. Unstimulated cells (none) served as control. **(A)** Representative contour plots of the expression of CD117 and IL-12Rβ2 on gated CD3^−^CD56^bright^ NK cells. Numbers indicate the percentage of CD117^+^ among IL-12Rβ2^−^ cells (rectangle). **(B)** Cumulative data of the percentage of CD117^+^ cells among IL-12Rβ2^−^ cells (*n* = 12). **(C,D)** Spearman correlation of CD117 expression with IFN-γ synthesis **(C)** and IL-12Rβ2 expression **(D)** on CD3^−^CD56^bright^ NK cells. **(E–G)** Recombinant IL-15 or SB431542 (inhibitor of the TGF-βRI) was added during stimulation of PBMC from trauma patients with *S. aureus* [the values for cells stimulated with *S. aureus* alone are also shown in Figures 1B,C and **(B)**]. **(E)** Percentage of CD117^+^ cells among IL-12Rβ2^−^CD3^−^CD56^bright^ NK cells (*n* = 11–12). **(F)** Expression of the IL-12Rβ2 chain on CD3^−^CD56^bright^ NK cells (*n* = 11–12). **(G)** Expression of IFN-γ in CD3^−^CD56^bright^ NK cells (*n* = 12–13). Horizontal lines indicate the median/interquartile range. Statistical differences between control and trauma **(B)** were tested using the Mann-Whitney U-test, differences between different culture conditions **(E–G)** were tested using the Wilcoxon signed rank test. *p < 0.05; **p < 0.01; and ***p < 0.001.

The expression of the IL-12Rβ2 chain is a check point in IFN-γ synthesis by NK cells after major trauma (19). In search of a potential mechanism that controls the expression of the IL-12Rβ2 chain we investigated how NK cells that do not express the IL-12Rβ2 (and therefore do not secrete IFN-γ) differ from IL-12Rβ2^+^cells. In order to induce IL-12Rβ2 expression PBMC from patients and controls were stimulated with *S. aureus*. There was a striking difference in terms of CD117 expression between IL-12Rβ2^+^ and IL-12Rβ2^−^ NK cells: the IL-12Rβ2 chain was almost exclusively expressed on CD117^−^ NK cells (Figure 3A). Further analysis of IL-12Rβ2^−^ NK cells revealed a two-fold increased percentage of CD117^+^ NK cells after major injury (Figure 3B). The expression of CD117 inversely correlated with the expression of the IL-12Rβ2 chain (Figure 3C) and with the production of IFN-γ (Figure 3D).

Next, the potential relationship between the expression of CD117 and NK cell function was examined. The expression of CD117 on NK cells is regulated by IL-15 in the environment (21). NK cell function after trauma is regulated by TGF-βRI signaling (19). Therefore, PBMC from injured patients were stimulated with *S. aureus* in the presence of recombinant IL-15, with an inhibitor of the TGF-βRI, or with the combination of both. Each component alone decreased the expression of CD117 on NK cells from injured patients. Even more effective was the combination of IL-15 with the TGF-βRI inhibitor (Figure 3E). Likewise, but in the inverse direction, the expression of the IL-12Rβ2 changed and increased by up to six-fold in the presence of IL-15 and the TGF-βRI inhibitor (Figure 3F). In contrast, only the combination of IL-15 with the TGF-βRI inhibitor enhanced the synthesis of IFN-γ (Figure 3G). Thus, IL-15 and inhibition of TGF-βRI signaling decrease the expression of CD117 that is associated with impaired NK cell function after trauma.

### T-bet Expression Is Reduced in NK Cells After Trauma

**Figure 4:**
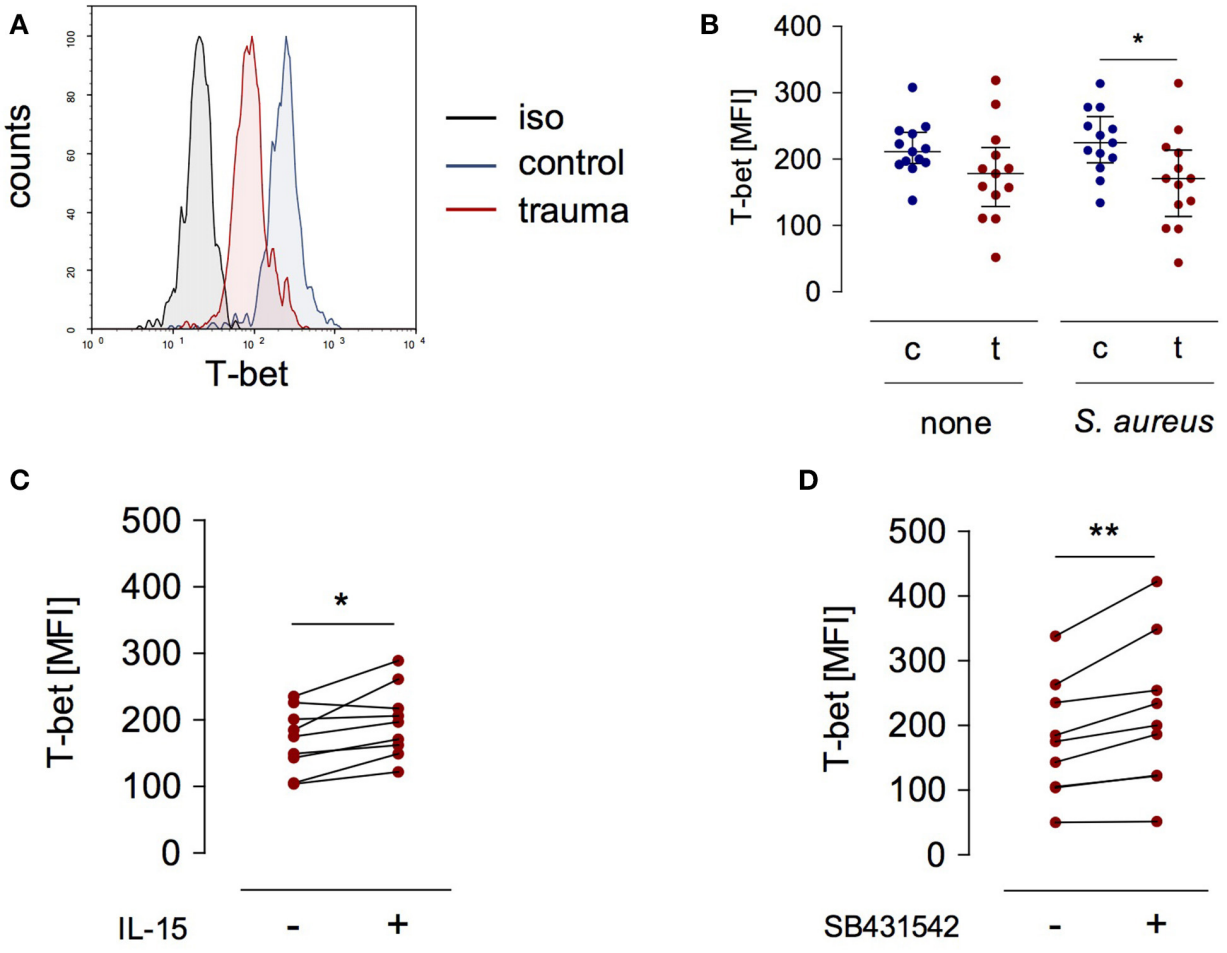
T-bet expression in stimulated CD56^bright^ NK cells is reduced after trauma. PBMC from control subjects (c) or patients (t) were stimulated with *S. aureus* and the expression of intracellular T-bet was examined. **(A)** Representative histogram of T-bet expression in gated CD3^−^CD56^bright^ NK cells. **(B)** Cumulative data of T-bet expression in CD56^bright^ NK cells (*n* = 13). **(C,D)** Recombinant IL-15 (**C**; *n* = 9) or SB431542 (**D**; *n* = 9) was added during stimulation of PBMC from trauma patients with *S. aureus* [the values for cells stimulated with *S. aureus* alone are also shown in **(B)**]. Horizontal lines indicate the median/interquartile range. Statistical differences between control and trauma **(B)** were tested using the Mann-Whitney *U*-test, differences between different culture conditions **(C,D)** were tested using the Wilcoxon signed rank test. *p < 0.05 and **p < 0.01. iso, isotype control.

T-bet is a relevant transcription factor that promotes *IL12RB2* and *IFNG* gene transcription in T lymphocytes and NK cells (12, 22). We examined the expression of T-bet in NK cells after severe injury and asked whether the expression of T-bet was altered in the presence of IL-15 or upon inhibition of the TGF-βRI. T-bet expression did not differ between NK cells from injured patients and healthy controls when analyzed *ex vivo* (Figure 1B). In contrast, after stimulation with *S. aureus*, NK cells from injured patients expressed less T-bet than NK cells from healthy subjects (Figures 4A,B). The expression of T-bet slightly increased in the presence of IL-15 (Figure 4C) or of the TGF-βRI inhibitor (Figure 4D). Thus, the changes of IL-12Rβ2 expression that are mediated by IL-15 and inhibition of the TGF-βRI in NK cells from injured patients are in part reflected by altered T-bet expression.

### Decreased Phosphorylation of mTOR Correlates With CD117 Expression

**Figure 5:**
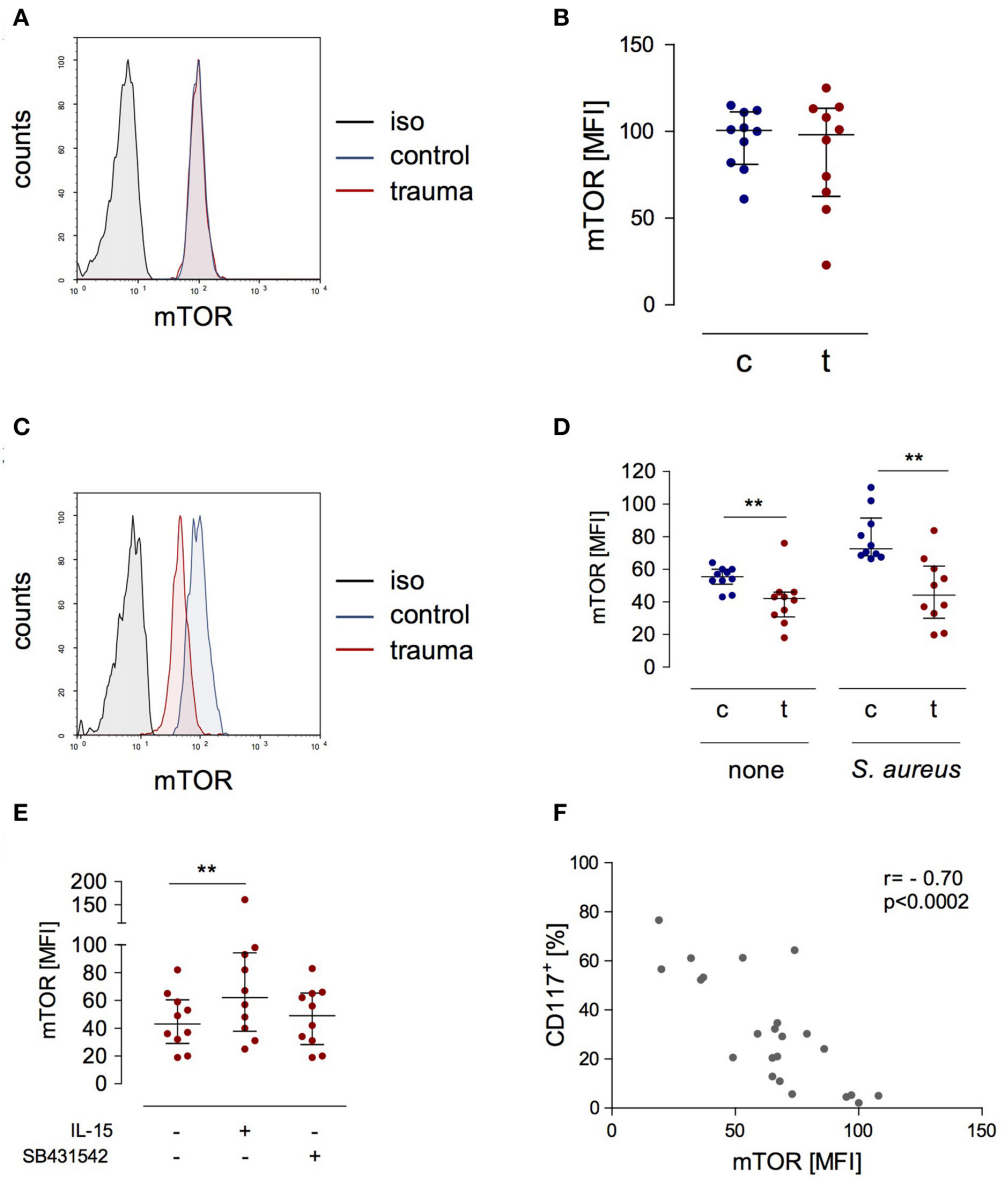
The expression of phosphorylated mTOR is reduced in stimulated CD56^bright^ NK cells after trauma and inversely correlates with the expression of CD117. **(A)** Representative histograms of phosphorylated mTOR expression in CD56^bright^ NK cells from control subjects (c) and trauma patients (t) *ex vivo*. **(B)** Cumulative data of phosphorylated mTOR expression *ex vivo* (*n* = 10). **(C,D)** PBMC were stimulated with *S. aureus* and intracellular expression of phosphorylated mTOR was determined after 18 h. Representative histograms after stimulation with *S. aureus* **(C)** and cumulative data (*n* = 10) of phosphorylated mTOR **(D)** are shown. **(E)** Recombinant IL-15 or SB431542 was added during stimulation of PBMC from trauma patients (*n* = 10) with *S. aureus* [the values for cells stimulated with *S. aureus* alone are also shown in **(D)**]. Horizontal lines indicate the median/interquartile range. Statistical differences between control and trauma **(B,D)** were tested using the Mann-Whitney *U*-test, differences between different culture conditions **(E)** were tested using the Wilcoxon signed rank test. **(F)** Spearman correlation between expression of CD117 and phosphorylated mTOR after stimulation with *S. aureus*. **p < 0.01. iso, isotype control.

A central molecule in the IL-15 signaling pathway during NK cell activation is “mammalian target of rapamycin” (mTOR) (23). We determined the expression of phosphorylated mTOR in NK cells after trauma and evaluated its potential regulation by IL-15 and inhibition of the TGF-βRI. There was no difference in mTOR phosphorylation in NK cells from injured patients and from healthy controls when analyzed *ex vivo* (Figures 5A,B). Stimulation with *S. aureus* strongly induced the phosphorylation of mTOR in NK cells from healthy controls but not in NK cells from trauma patients (Figures 5C,D). The presence of IL-15 during stimulation with *S. aureus* increased the phosphorylation of mTOR in NK cells from trauma patients while the inhibition of the TGF-βRI remained without consequences (Figure 5E). The pattern of mTOR phosphorylation resembled the changes in CD117 expression on NK cells (Figure 3E) though in the opposite direction. Indeed, there is a negative correlation between mTOR and CD117 (Figure 5F). Thus, mTOR phosphorylation in NK cells after severe injury is reduced and inversely correlates with CD117 expression.

### mTOR Phosphorylation in NK Cells Promotes the Synthesis of IFN-γ

**Figure 6:**
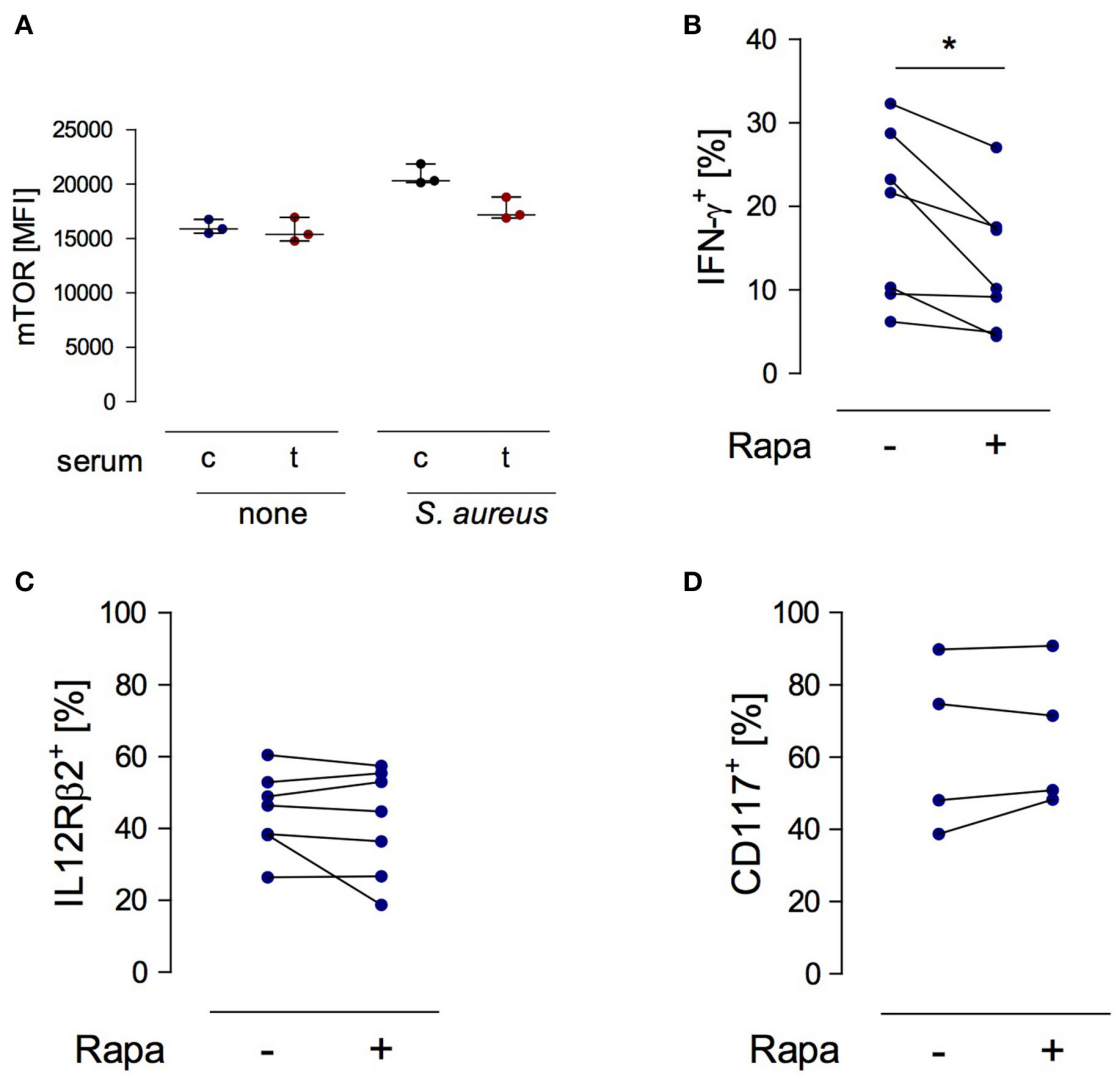
mTOR is a cell-intrinsic regulator of IFN-γ synthesis in NK cells exposed to *S. aureus*. **(A)** Cultures of PBMC from healthy donors (*n* = 3) were set up in serum from control subjects (c) or from trauma patients (t) and were stimulated with *S. aureus*. Unstimulated cells served as control (none). The mean fluorescence intensity (MFI) of phosphorylated mTOR was determined in gated CD3^−^CD56^bright^ NK cells after 18 h. Horizontal lines indicate the median/interquartile range. **(B–D)** Purified NK cells from healthy donors (*n* = 2–4) were exposed to conditioned medium of PBMC from healthy donors that had been obtained after stimulation with *S. aureus* (2–3 different batches). Rapamycin (rapa) was added or not (–). The percentage of CD56^bright^ NK cells positive for IFN-γ **(B)**, IL-12Rβ2 **(C)**, and CD117 **(D)** was quantified. Statistical differences were tested using the Wilcoxon signed rank test. *p < 0.05.

Considering the suppressive activity of serum from injured patients on the function of NK cells (19) the question arose whether any factors in the serum interfere with mTOR phosphorylation in NK cells. To address this issue, PBMC from healthy subjects were stimulated with *S. aureus* in the presence of serum from trauma patients or from healthy controls. The basal level of mTOR phosphorylation in NK cells was not affected by serum from injured patients but tended to decrease upon stimulation with *S. aureus* (Figure 6A).

In order to examine the relevance of mTOR in NK cell function we established a cell culture system with purified NK cells. Purified NK cells do not respond to *S. aureus* since they require the interaction with monocytes or DCs as source of IL-12 (24). Therefore, isolated NK cells from healthy donors were cultured in conditioned cell-free medium obtained from PBMC after stimulation with *S. aureus*. Rapamycin was used to inhibit mTOR activity. The presence of rapamycin diminished the production of IFN-γ (Figure 6B) by NK cells but did not affect the expression of the IL-12Rβ2 chain (Figure 6C). Furthermore, inhibition of mTOR did not change the expression of CD117 on NK cells (Figure 6D). Thus, mTOR is an intrinsic regulator of IFN-γ synthesis in NK cells in the context of *S. aureus* infection.

## Discussion

Immediately after major injury, circulating NK cells display a long-lasting impaired capacity to produce IFN-γ in response to microbial stimuli (19). While soluble factors in the serum mediate NK cell suppression early after injury, cell-intrinsic changes in NK cells are responsible for the anergic state late after trauma that renders NK cell unresponsive to otherwise stimulatory cytokines such as IL-12 and IL-2 (19). NK cells do not show signs of activation such as CD25 and CD69 after injury (19) nor differed in the expression of various characteristic markers of mature NK cells from cells of control subjects (except a reduced expression of CD117). Thus, the impaired function of NK cells after severe injury is not reflected by an altered phenotype at least according to the markers that we have examined so far. According to a recent study, CD39 is differentially expressed on NK cells after trauma (25) and might be a candidate for phenotyping NK cells from trauma patients.

The striking modulation of NK cells after major injury was only visible when the cells were exposed to *S. aureus* that mimicked an infectious challenge. This finding indicates that severe injury “primes” NK cells for an altered responsiveness to infectious agents. We propose that major injury induces a functional reprogramming of NK cells that is responsible for the impaired capacity of the cells to secrete IFN-γ while their cytotoxic function remains unaffected (19).

Our previous work has shown that the cell-intrinsic inhibition of NK cells requires 8 day to be fully established (19). Here, we provide first evidence that CD117 and mTOR are potential key molecules in the development of NK cell dysregulation after trauma. The expression of CD117 inversely correlated with the expression of IL-12Rβ2 and IFN-γ. This finding points to a potential inhibitory effect of the CD117-induced signaling pathway on the IL-12Rβ2/IFN-γ axis. Cell type-specific differences in the biological effect of CD117 signaling have been reported: in mast cells the activation of the CD117 tyrosine kinase triggers PI3K, MAPK, and JAK/STAT pathways and thereby induces the release of pro-inflammatory cytokines and histamine (26). In DCs, signaling through CD117 induces the secretion of IL-6 through PI3K activation (27). Some of these CD117-induced pathways overlap with IL-12Rβ2 signaling (28). Since CD117-induced signaling in NK cells has not been addressed so far it remains speculative whether and how CD117 interferes with IL-12-induced IFN-γ synthesis in NK cells.

We observed a striking inverse correlation of CD117 expression with the phosphorylation of mTOR in NK cells late after trauma. mTOR is an intracellular serine/threonine kinase and plays a central role in cytokine secretion, survival, and proliferation through its role as master switch in cell metabolism. CD117 signaling has been described to regulate mTOR phosphorylation in mesenchymal stem cells (29). A similar regulatory function of CD117 on mTOR might take place in NK cells and explain the inverse correlation of CD117 and mTOR. Detailed analyses of signaling pathways in NK cells are required in future to prove this assumption of such a functional relationship between CD117 and mTOR.

Previous studies have shown that mTOR is critical for the maintenance of the cytotoxic activity and for metabolic control of NK cells but not for their IFN-γ secretion in response to recombinant cytokines (23, 30, 31). We established a novel *in vitro* model that mimicked the activation of NK cells as it takes place during stimulation of PBMC with *S. aureus* but that was independent from recombinant cytokines and from accessory cells such as monocytes. We provide evidence that under certain circumstances mTOR indeed promotes the synthesis of IFN-γ by NK cells. However, so far we could not identify the mediators that are responsible for mTOR activation in NK cells in our *in vitro* model. Based on these findings we assume that reduced phosphorylation of mTOR in NK cells late after trauma and signaling through the TGF-βRI both contribute to their impaired capacity to produce IFN-γ in response to *S. aureus*.

In contrast to IFN-γ synthesis, the inhibition of mTOR in NK cells did not affect the expression of CD117. Thus, it is unlikely that the expression of CD117 is directly regulated by mTOR in NK cells.

It has been described that Smad1/5/8, components of the signaling pathway downstream of the TGF-β receptor, promote the expression of CD117 in primordial follicles (32). In line, we detected decreased CD117 expression on NK cells from injured patients upon inhibition of the TGF-βRI. We have previously shown that growth/differentiation factor (GDF) 15 is present in the serum at high levels after major trauma, signals through the TGF-βRI and activates Smad1/5/8 (19). Accordingly, the increased expression of CD117 on NK cells after major injury might be mediated by circulating GDF-15. In contrast, the inhibition of TGF-βRI signaling did not affect the phosphorylation of mTOR in NK cells of the patients. This finding further supports our assumption that CD117 is not under control of mTOR.

In line with a previous study (21), we observed that the expression of CD117 on NK cells decreased when recombinant IL-15 was added during stimulation with *S. aureus*. Maximal reduction of CD117 expression was achieved with a combination of IL-15 and inhibition of the TGF-βRI that was at the same time the most effective in upregulation of the IL-12Rβ2 chain. Interestingly, IL-12Rβ2 was only expressed on CD117^−^ NK cells. This finding implies that the signaling pathways induced by CD117 and TGF-βRI cooperate and prevent the expression of the IL-12Rβ2 chain. Certainly, additional studies are required to confirm the existence of this novel cross-talk between CD117 and TGF-βRI in NK cells.

The synthesis of IFN-γ by NK cells correlated with the expression of the IL-12Rβ2 chain. Unexpectedly, addition of IL-15 or inhibition of the TGF-βRI did not result in increased IFN-γ synthesis by NK cells after major trauma despite augmented expression of the IL-12Rβ2 chain. This is in contrast to the well-known capacity of IL-15 to enhance IFN-γ secretion by NK cells from healthy subjects (33). NK cells from trauma patients expressed the IL-15 receptor subunits at levels comparable to NK cells from controls. This finding argues against a reduced sensing of IL-15 as the origin of the unchanged IFN-γ synthesis. But IL-15 was efficient in amplifying the production of IFN-γ when it was combined with the inhibitor of the TGF-βRI inhibitor. We suggest that yet unknown mechanisms exist in NK cells of severely injured patients that interfere with the signaling pathway downstream of the IL-12Rβ2 chain and that are regulated by IL-15- and TGF-βRI-induced signaling. In this regard, the transcription factor T-bet might be of relevance as it was reduced after trauma and increased in response to IL-15 and inhibition of the TGF-βRI.

Increased mTOR activity is a central component in the development of so called “trained immunity” that describes the long-term increased response to a secondary stimulus after an acute, often infectious insult (34). Trained immunity of NK cells is a consequence of cytomegalo virus infection and is associated with enhanced cytotoxicity in response to repeated infection (35). Defective trained immunity of monocytes occurs during sepsis and is considered to enhance the risk for secondary infections (36). Considering the reduced mTOR phosphorylation and the relevance of IFN-γ in immune defense we hypothesize that NK cells undergo an impaired trained immunity after major traumatic injury that, in case of a subsequent infectious insult, may result in a disturbed NK cell function. Due to the small sample size of our pilot study we could not differentiate NK cell function between patients who later developed infectious complications and those who remained free of an infection to prove this hypothesis.

In conclusion, there exists an inverse relationship between CD117 and phosphorylated mTOR in CD56^bright^ NK cells after exposure to *S. aureus*. After trauma, this relationship is shifted toward CD117 and is associated with a disturbed IL-12/IFN-γ axis. Restoration of the capacity for mTOR phosphorylation by application of IL-15 in combination with inhibition of the TGF-βRI signaling pathway might represent a potential therapeutic option to improve the function of NK cells after major trauma.

## Data Availability Statement

All datasets generated for this study are included in the article/Supplementary Material.

## Ethics Statement

The studies involving human participants were reviewed and approved by the ethics committee of the University Hospital Essen. The patients/participants provided their written informed consent to participate in this study.

## Author Contributions

BB and MH-S designed and performed the experiments, analyzed and interpreted the data, and wrote the manuscript. SV and MD provided material of the patients and contributed to the study design. SF supervised the study, designed the experiments, analyzed and interpreted the data, and wrote the manuscript. All authors contributed to the article and approved the submitted version.

## Conflict of Interest

The authors declare that the research was conducted in the absence of any commercial or financial relationships that could be construed as a potential conflict of interest.

## References

[1] Mira JC Cuschieri J Ozrazgat-Baslanti T Wang Z Ghita GL Loftus TJ. The epidemiology of chronic critical illness after severe traumatic injury at two level-one trauma centers. Crit Care Med. (2017) 45:1989–96. 10.1097/CCM.0000000000002697 28837430 PMC5693769

[2] Lord JM Midwinter MJ Chen YF Belli A Brohi K Kovacs EJ. The systemic immune response to trauma: an overview of pathophysiology and treatment. Lancet. (2014) 384:1455–65. 10.1016/S0140-6736(14)60687-5 25390327 PMC4729362

[3] Kimura F Shimizu H Yoshidome H Ohtsuka M Miyazaki M. Immunosuppression following surgical and traumatic injury. Surg Today. (2010) 40:793–808. 10.1007/s00595-010-4323-z 20740341 PMC7101797

[4] Horiguchi H Loftus TJ Hawkins RB Raymond SL Stortz JA Hollen MK. Innate immunity in the persistent inflammation, immunosuppression, and catabolism syndrome and its implications for therapy. Front Immunol. (2018) 9:595. 10.3389/fimmu.2018.00595 29670613 PMC5893931

[5] Lodoen MB Lanier LL. Natural killer cells as an initial defense against pathogens. Curr Opin Immunol. (2006) 18:391–398. 10.1016/j.coi.2006.05.002 16765573 PMC7127478

[6] Caligiuri MA. Human natural killer cells. Blood. (2008) 112:461–9. 10.1182/blood-2007-09-077438 18650461 PMC2481557

[7] Poli A Michel T Theresine M Andres E Hentges F Zimmer J. CD56bright natural killer (NK) cells: an important NK cell subset. Immunology. (2009) 126:458–65. 10.1111/j.1365-2567.2008.03027.x 19278419 PMC2673358

[8] Michel T Hentges F Zimmer J. Consequences of the crosstalk between monocytes/macrophages and natural killer cells. Front Immunol. (2013) 3:403. 10.3389/fimmu.2012.00403 23316194 PMC3539656

[9] Thomas R Yang X. NK-DC crosstalk in immunity to microbial infection. J Immunol Res. (2016) 2016:6374379. 10.1155/2016/6374379 28097157 PMC5206438

[10] Thierfelder WE van Deursen JM Yamamoto K Tripp RA Sarawar SR Carson RT. Requirement for Stat4 in interleukin-12-mediated responses of natural killer and T cells. Nature. (1996) 382:171–4. 10.1038/382171a0 8700208

[11] Jacobson NG Szabo SJ Weber-Nordt RM Zhong Z Schreiber RD Darnell JE., Interleukin 12 signaling in T helper type 1 (Th1) cells involves tyrosine phosphorylation of signal transducer and activator of transcription (Stat)3 and Stat4. J Exp Med. (1995) 181:1755–62. 10.1084/jem.181.5.1755 7722452 PMC2191986

[12] Afkarian M Sedy JR Yang J Jacobson NG Cereb N Yang SY. T-bet is a STAT1-induced regulator of IL-12R expression in naive CD4+ T cells. Nat Immunol. (2002) 3:549–57. 10.1038/ni794 12006974

[13] Wang KS Frank DA Ritz J. Interleukin-2 enhances the response of natural killer cells to interleukin-12 through up-regulation of the interleukin-12 receptor and STAT4. Blood. (2000) 95:3183–90. 10.1182/blood.V95.10.3183.010k36_3183_3190 10807786

[14] Lucas M Schachterle W Oberle K Aichele P Diefenbach A. Dendritic cells prime natural killer cells by trans-presenting interleukin 15. Immunity. (2007) 26:503–17. 10.1016/j.immuni.2007.03.006 17398124 PMC2084390

[15] Zhang J Marotel M Fauteux-Daniel S Mathieu AL Viel S Marcais A. T-bet and eomes govern differentiation and function of mouse and human NK cells and ILC1. Eur J Immunol. (2018) 48:738–50. 10.1002/eji.201747299 29424438

[16] Freud AG Yokohama A Becknell B Lee MT Mao HC Ferketich AK. Evidence for discrete stages of human natural killer cell differentiation *in vivo*. J Exp Med. (2006) 203:1033–43. 10.1084/jem.20052507 16606675 PMC2118285

[17] Matos ME Schnier GS Beecher MS Ashman LK William DE Caligiuri MA. Expression of a functional c-kit receptor on a subset of natural killer cells. J Exp Med. (1993) 178:1079–84. 10.1084/jem.178.3.1079 7688785 PMC2191187

[18] Seshadri A Brat GA Yorkgitis BK Keegan J Dolan J Salim A. Phenotyping the immune response to trauma: a multiparametric systems immunology approach. Crit Care Med. (2017) 45:1523–30. 10.1097/CCM.0000000000002577 28671900 PMC10114604

[19] Kleinertz H Hepner-Schefczyk M Ehnert S Claus M Halbgebauer R Boller L. Circulating growth/differentiation factor 15 is associated with human CD56(bright) natural killer cell dysfunction and nosocomial infection in severe systemic inflammation. EBioMedicine. (2019) 43:380–91. 10.1016/j.ebiom.2019.04.018 30992245 PMC6557805

[20] Abel AM Yang C Thakar MS Malarkannan S. Natural killer cells: development, maturation, and clinical utilization. Front Immunol. (2018) 9:1869. 10.3389/fimmu.2018.01869 30150991 PMC6099181

[21] Pradier A Tabone-Eglinger S Huber V Bosshard C Rigal E Wehrle-Haller B. Peripheral blood CD56(bright) NK cells respond to stem cell factor and adhere to its membrane-bound form after upregulation of c-kit. Eur J Immunol. (2014) 44:511–520. 10.1002/eji.201343868 24150691

[22] Szabo SJ Sullivan BM Stemmann C Satoskar AR Sleckman BP Glimcher LH. Distinct effects of T-bet in TH1 lineage commitment and IFN-gamma production in CD4 and CD8 T cells. Science. (2002) 295:338–42. 10.1126/science.1065543 11786644

[23] Marcais A Cherfils-Vicini J Viant C Degouve S Viel S Fenis A. The metabolic checkpoint kinase mTOR is essential for IL-15 signaling during the development and activation of NK cells. Nat Immunol. (2014) 15:749–57. 10.1038/ni.2936 24973821 PMC4110708

[24] Haller D Serrant P Granato D Schiffrin EJ Blum S. Activation of human NK cells by staphylococci and lactobacilli requires cell contact-dependent costimulation by autologous monocytes. Clin Diagn Lab Immunol. (2002) 9:649–57. 10.1128/CDLI.9.3.649-657.2002 11986274 PMC119993

[25] Seshadri A Brat GA Yorkgitis BK Giangola M Keegan J Nguyen JP. Altered monocyte and NK cell phenotypes correlate with posttrauma infection. J Trauma Acute Care Surg. (2019) 87:337–41. 10.1097/TA.0000000000002264 31008865

[26] Reber L Da Silva CA Frossard N. Stem cell factor and its receptor c-Kit as targets for inflammatory diseases. Eur J Pharmacol. (2006) 533:327–40. 10.1016/j.ejphar.2005.12.067 16483568

[27] Krishnamoorthy N Oriss TB Paglia M Fei M Yarlagadda M Vanhaesebroeck B. Activation of c-Kit in dendritic cells regulates T helper cell differentiation and allergic asthma. Nat Med. (2008) 14:565–73. 10.1038/nm1766 18454155 PMC3664066

[28] Watford WT Hissong BD Bream JH Kanno Y Muul L O'Shea JJ. Signaling by IL-12 and IL-23 and the immunoregulatory roles of STAT4. Immunol Rev. (2004) 202:139–56. 10.1111/j.0105-2896.2004.00211.x 15546391

[29] Lee Y Jung J Cho KJ Lee SK Park JW Oh IH. Increased SCF/c-kit by hypoxia promotes autophagy of human placental chorionic plate-derived mesenchymal stem cells via regulating the phosphorylation of mTOR. J Cell Biochem. (2013) 114:79–88. 10.1002/jcb.24303 22833529

[30] Morgan DJ Davis DM. Distinct effects of dexamethasone on human natural killer cell responses dependent on cytokines. Front Immunol. (2017) 8:432. 10.3389/fimmu.2017.00432 28450865 PMC5389971

[31] Keating SE Zaiatz-Bittencourt V Loftus RM Keane C Brennan K Finlay DK. Metabolic reprogramming supports IFN-gamma production by CD56bright NK cells. J Immunol. (2016) 196:2552–60. 10.4049/jimmunol.1501783 26873994

[32] Ding X Zhang X Mu Y Li Y Hao J. Effects of BMP4/SMAD signaling pathway on mouse primordial follicle growth and survival via up-regulation of Sohlh2 and c-kit. Mol Reproduct Dev. (2013) 80:70–8. 10.1002/mrd.22138 23212987

[33] Ali AK Nandagopal N Lee SH. IL-15-PI3K-AKT-mTOR: a critical pathway in the life journey of natural killer cells. Front Immunol. (2015) 6:355. 10.3389/fimmu.2015.00355 26257729 PMC4507451

[34] Netea MG Joosten LA Latz E Mills KH Natoli G Stunnenberg HG. Trained immunity: a program of innate immune memory in health and disease. Science. (2016) 52: aaf1098. 10.1126/science.aaf1098 27102489 PMC5087274

[35] Sun JC Madera S Bezman NA Beilke JN Kaplan MH Lanier LL. Proinflammatory cytokine signaling required for the generation of natural killer cell memory. J Exp Med. (2012) 209:947–954. 10.1084/jem.20111760 22493516 PMC3348098

[36] Cheng SC Scicluna BP Arts RJ Gresnigt MS Lachmandas E Giamarellos-Bourboulis EJ. s Broad defects in the energy metabolism of leukocytes underlie immunoparalysis in sepsis. Nat Immunol. (2016) 17:406–13. 10.1038/ni.3398 26950237

