# Supplementary material for: An Inverse Relationship Between c-Kit/CD117 and mTOR Confers NK Cell Dysregulation Late After Severe Injury
Source: Front Immunol. 2020 Jun 25;11:1200. doi: 10.3389/fimmu.2020.01200 (PMC7330140; doi:10.3389/fimmu.2020.01200)
Supplement: Supplementary file 1 [file Table_1.DOCX]

**Supplementary Table 1.** Patient characteristics

|  | **Control** | **Trauma** |
| --- | --- | --- |
|  | n=14 | n=14 |
| Age | 52 (40-66) | 54 (40-70) |
| Sex (f/m) | 6/8 | 6/8 |
| Injury severity score (ISS) |  | 22 (18-27) |
| SOFA (day 8) |  | 4 (1-8) |
| ICU length of stay |  | 15 (8-44) |
| Hospital length of stay |  | 25 (15-55) |
| Sepsis |  | 21% |
| Sepsis diagnosis day |  | 9 (8-22) |
| leukocytes/nl _Day8_ |  | 11 (9 - 14) |
| CRP (day 8) [mg/dl] |  | 13 (10 -18) |
| PCT (day 8) [μg/L] |  | 0,2 (0,1 - 0,7) |

Values are expressed as median (interquartile range). SOFA, sequential organ failure assessment; ICU, intensive care unit; CRP,C-reactive protein; PCT, procalcitonin
