# Supplementary material for: An Inverse Relationship Between c-Kit/CD117 and mTOR Confers NK Cell Dysregulation Late After Severe Injury
Source: Front Immunol. 2020 Jun 25;11:1200. doi: 10.3389/fimmu.2020.01200 (PMC7330140; doi:10.3389/fimmu.2020.01200)
Supplement: Supplementary file 2 [file Presentation_1.PPTX]

## Slide 1
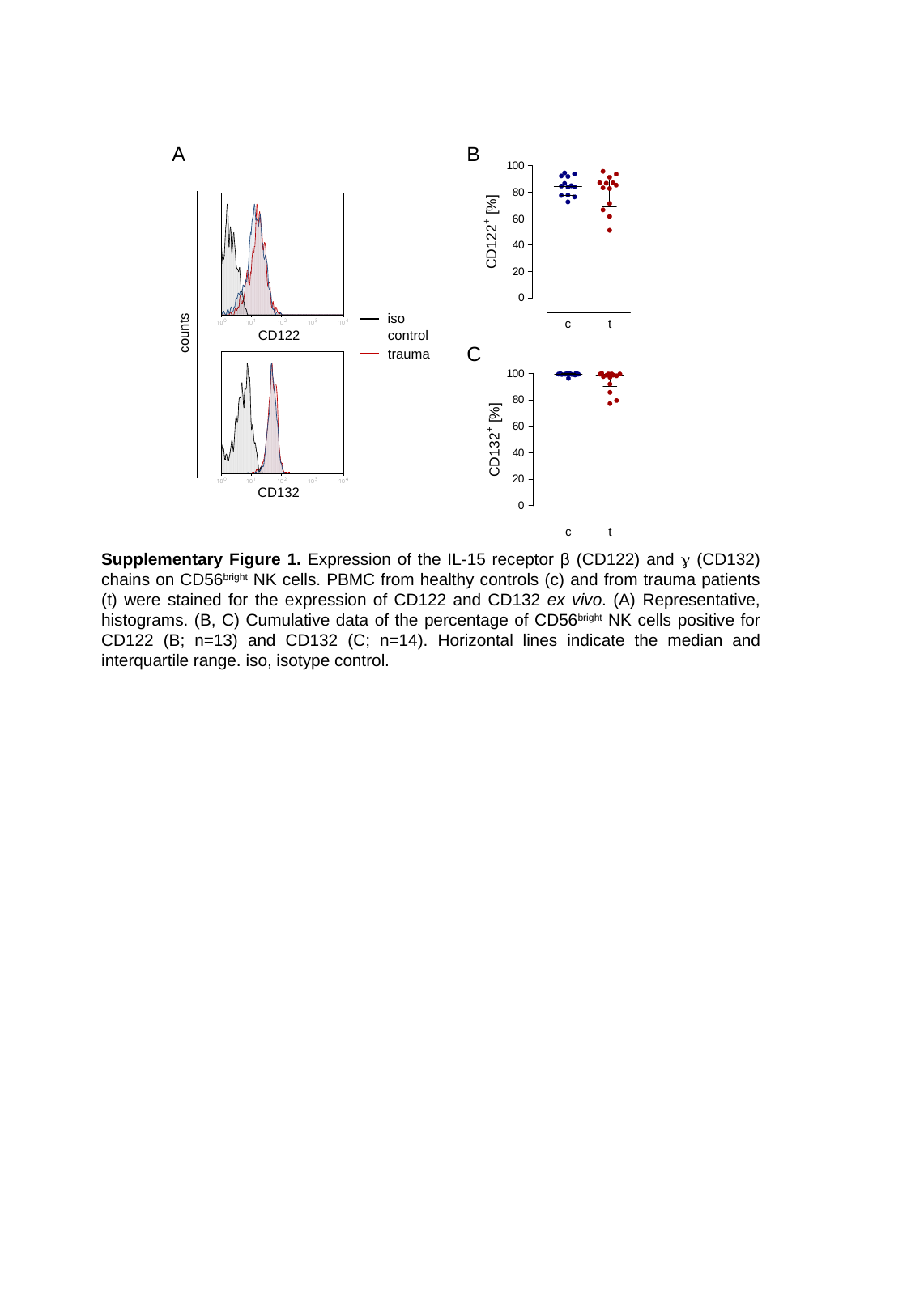

A
B
iso
counts
CD122
control
C
trauma
CD132
Supplementary Figure 1. Expression of the IL-15 receptor β (CD122) and g (CD132) chains on CD56bright NK cells. PBMC from healthy controls (c) and from trauma patients (t) were stained for the expression of CD122 and CD132 ex vivo. (A) Representative, histograms. (B, C) Cumulative data of the percentage of CD56bright NK cells positive for CD122 (B; n=13) and CD132 (C; n=14). Horizontal lines indicate the median and interquartile range. iso, isotype control.
